# Variability in Ejection Fraction Measured By Echocardiography, Gated Single-Photon Emission Computed Tomography, and Cardiac Magnetic Resonance in Patients With Coronary Artery Disease and Left Ventricular Dysfunction

**DOI:** 10.1001/jamanetworkopen.2018.1456

**Published:** 2018-08-31

**Authors:** Patricia A. Pellikka, Lilin She, Thomas A. Holly, Grace Lin, Padmini Varadarajan, Ramdas G. Pai, Robert O. Bonow, Gerald M. Pohost, Julio A. Panza, Daniel S. Berman, David L. Prior, Federico M. Asch, Salvador Borges-Neto, Paul Grayburn, Hussein R. Al-Khalidi, Karol Miszalski-Jamka, Patrice Desvigne-Nickens, Kerry L. Lee, Eric J. Velazquez, Jae K. Oh

**Affiliations:** 1 Department of Cardiovascular Medicine, Mayo Clinic, Rochester, Minnesota; 2 Duke Clinical Research Institute, Durham, North Carolina; 3 Division of Cardiology, Department of Medicine, Northwestern University Feinberg School of Medicine, Chicago, Illinois; 4 Department of Medicine, Loma Linda University, Loma Linda, California; 5 Department of Cardiology, Loma Linda University, Loma Linda, California; 6 Department of Medicine, Riverside School of Medicine, University of California, Riverside; 7 Department of Cardiology, Riverside School of Medicine, University of California, Riverside; 8 Westchester Medical Center, New York Medical College, Valhalla; 9 Cedars-Sinai Medical Center, Los Angeles, California; 10 Department of Cardiology, St Vincent’s Hospital, University of Melbourne, Melbourne, Australia; 11 Department of Medicine, St Vincent’s Hospital, University of Melbourne, Melbourne, Australia; 12 Section of Interventional Cardiology, MedStar Washington Hospital Center, Washington, DC; 13 Division of Nuclear Medicine, Department of Radiology, Duke University School of Medicine, Durham, North Carolina; 14 Division of Cardiology, Department of Medicine, Duke Clinical Research Institute, Durham, North Carolina; 15 Cardiology Section, Department of Internal Medicine, Baylor University Medical Center, Dallas, Texas; 16 Department of Biostatistics and Bioinformatics, Duke Clinical Research Institute, Durham, North Carolina; 17 Division of Magnetic Resonance Imaging, Silesian Center for Heart Diseases, Zabrze, Poland; 18 Division of Cardiovascular Sciences, National Heart, Lung, and Blood Institute, National Institutes of Health, Bethesda, Maryland

## Abstract

**Importance:**

Clinical decisions are frequently based on measurement of left ventricular ejection fraction (LVEF). Limited information exists regarding inconsistencies in LVEF measurements when determined by various imaging modalities and the potential impact of such variability.

**Objective:**

To determine the intermodality variability of LVEF measured by echocardiography, gated single-photon emission computed tomography (SPECT), and cardiovascular magnetic resonance (CMR) in patients with left ventricular dysfunction.

**Design, Setting, and Participants:**

International multicenter diagnostic study with LVEF imaging performed at 127 clinical sites in 26 countries from July 24, 2002, to May 5, 2007, and measured by core laboratories. Secondary study of clinical diagnostic measurements of LVEF in the Surgical Treatment for Ischemic Heart Failure (STICH), a randomized trial to identify the optimal treatment strategy for patients with LVEF of 35% or less and coronary artery disease. Data analysis was conducted from March 19, 2016, to May 29, 2018.

**Main Outcomes and Measures:**

At baseline, most patients had an echocardiogram and subsets of patients underwent SPECT and/or CMR. Left ventricular ejection fraction was measured by a core laboratory for each modality independent of the results of other modalities, and measurements were compared among imaging methods using correlation, Bland-Altman plots, and coverage probability methods. Association of LVEF by each method and death was assessed.

**Results:**

A total of 2032 patients (mean [SD] age, 60.9 [9.6] years; 1759 [86.6%] male) with baseline LVEF data were included. Correlation of LVEF between modalities was *r* = 0.601 (for biplane echocardiography and SPECT [n = 385]), *r* = 0.493 (for biplane echocardiography and CMR [n = 204]), and *r* = 0.660 (for CMR and SPECT [n = 134]). Bland-Altman plots showed only moderate agreement in LVEF measurements from all 3 core laboratories with no substantial overestimation or underestimation of LVEF by any modality. The percentage of observations that fell within a range of 5% ranged from 43% to 54% between different imaging modalities.

**Conclusions and Relevance:**

In this international multicenter study of patients with coronary artery disease and reduced LVEF, there was substantial variation between modalities in LVEF determination by core laboratories. This variability should be considered in clinical management and trial design.

**Trial Registration:**

Clinicaltrials.gov Identifier: NCT00023595

## Key Points

**Question:**

What is the variability in left ventricular ejection fraction (LVEF) as measured by different cardiac imaging modalities?

**Findings:**

In this multicenter diagnostic study of 2032 patients with coronary artery disease and LVEF of 35% or less with imaging interpreted by core laboratories, correlation of LVEF between modalities ranged from *r* = 0.493 (for biplane echocardiography and cardiovascular magnetic resonance) to *r* = 0.660 (for cardiovascular magnetic resonance and gated single-photon emission computed tomography). There was no systematic overestimation or underestimation of LVEF for any modality.

**Meaning:**

There is substantial variability in LVEF assessment between modalities, which should be considered in trial design and clinical management.

## Teaser

This multicenter diagnostic study determines the intermodality variability of left ventricular ejection fraction (LVEF) measured by echocardiography, gated single-photon emission computed tomography (SPECT), and cardiovascular magnetic resonance (CMR) in patients with coronary artery disease and left ventricular dysfunction.

## Introduction

Feasible, accurate, and reproducible assessment of left ventricular ejection fraction (LVEF) is an important objective of noninvasive cardiac imaging. Whether LVEF is preserved or reduced currently forms the basis for the classification of patients with heart failure. Additionally, LVEF is an important predictor of prognosis in patients with myocardial infarction,^1,2,3^ heart failure,^4,5,6^ and valve disease.^7^ Moreover, current practice guidelines use LVEF thresholds for decision making in different clinical scenarios, such as the recommendation regarding device implantation or pharmacologic therapy in patients with heart failure^8,9^ and the recommendation for valve replacement in patients with severe valvular heart disease.^10^ Left ventricular ejection fraction is also a common enrollment criterion and/or end point for clinical trials.^11^

Left ventricular ejection fraction can be determined by using multiple noninvasive imaging modalities, including echocardiography, cardiac magnetic resonance (CMR) imaging, and gated single-photon emission computed tomography (SPECT) imaging. All of these methods are routinely used for clinical decision making as well as research study enrollment. However, few data exist regarding the agreement between LVEF determined by these different methods. Prior studies have been limited by small numbers of participants, sometimes including only healthy volunteers, with imaging performed by a single center, and have compared only 2 imaging modalities.^12,13,14^ As LVEF cut points are often the basis for clinical management decisions and trial eligibility, the implications of variability are substantial.

The Surgical Treatment for Ischemic Heart Failure (STICH) was an international multicenter trial aimed to compare coronary artery bypass grafting (CABG) and medical therapy for patients with heart failure, coronary artery disease (CAD), and left ventricular (LV) systolic dysfunction defined as LVEF of 35% or less.^15,16^ In this trial, any of 3 diagnostic methods (echocardiography, gated SPECT imaging, or CMR) could be used by a local clinical site to measure LVEF in order to determine a patient’s trial eligibility. All patients enrolled in the STICH trial were required to have a baseline determination of LVEF, and a subset of patients underwent this determination by multiple modalities, including echocardiography, gated SPECT imaging, and/or CMR. All LVEF data obtained by echocardiography, CMR, and SPECT were measured by respective core laboratories. Therefore, the STICH trial provides a unique opportunity to correlate core laboratory assessment of LVEF data between different modalities.

We conducted this study to determine the variability among imaging modalities and among different echocardiographic methods for assessing LVEF in patients with reduced LV systolic function, and to compare the association between these measurements and subsequent mortality in patients with ischemic cardiomyopathy.

## Methods

### Patients

A total of 2136 patients with LVEF of 35% or less were enrolled in the STICH trial.^16^ All patients provided written informed consent, as approved by the local institutional review board. Patients were enrolled at 127 clinical sites in 26 countries from July 24, 2002, to May 5, 2007; all had CAD amenable to CABG. Each of the 127 enrolling sites had to obtain institutional review board approval for STICH. We followed the Standards for Reporting of Diagnostic Accuracy (STARD) reporting guideline. A manual of operation for each modality was produced by the core laboratory for that modality to standardize imaging technique. Each site was required to submit 1 to 3 studies that fulfilled imaging requirements before enrollment. When studies did not meet these requirements, additional studies were requested until requirements were met. Patients with baseline imaging data received by the core laboratories for 1 or more imaging modalities were considered for inclusion. Patients with LVEF measured 90 days or more from study randomization or with study quality deemed by the core laboratory as being unusable for measurement were excluded. Determination of LVEF was performed by a separate core laboratory for each modality, independent of clinical information, treatment assignment, and data from other modalities.^17^ Each core laboratory provided oversight of quality control and assessed the quality of each study as excellent, good, fair, borderline, or unusable. Left ventricular end-systolic volume for each modality was indexed for body surface area.

### Calculation of LVEF

Left ventricular ejection fraction was determined from LV end-diastolic volume and end-systolic volume using the following standard formula:

LVEF = [(end-diastolic volume) – (end-systolic volume)]/end-diastolic volume

### Imaging

Echocardiography was performed at most sites for patient enrollment. Determination of LVEF was attempted according to the recommendations of the American Society of Echocardiography^18^ using the Simpson method.^17^ Measurements were averaged over 3 cardiac cycles for patients in sinus rhythm, and 3 to 5 cardiac cycles for those in atrial fibrillation. If 2 apical views were not available for LV volume measurement, only 1 apical view (single-plane Simpson measurement) was used for determination of LVEF. For Simpson measurements, the LV endocardial border was traced contiguously from one side of the mitral annulus to the other side excluding the papillary muscles and trabeculations. Left ventricular ejection fraction was also estimated visually in most patients and when the definition of the LV endocardial border was not satisfactory from any of the apical views, visual estimate was the only echocardiographic determination of LVEF.^19^

Gated myocardial perfusion SPECT imaging, predominantly using sestamibi, was performed at clinical sites using a standard protocol. The gated raw projections were reconstructed by the radionuclide core laboratory using automatic software (AutoSPECT). When appropriate, an algorithm was applied to correct for motion. Resting studies accounted for 82% of measurements of LVEF; the remainder was obtained from poststress studies. Gated short-axis images were reviewed by a core laboratory technologist to optimize the accuracy of automatically determined LV contours, which were measured in end-systole and end-diastole. Manual adjustment addressed incorrect valve plane placement or contour deviations because of extracardiac radioactivity. Gated SPECT images were analyzed for LVEF by the radionuclide core laboratory using quantitative gated SPECT (QGS) software.^20^

Cardiac magnetic resonance imaging was performed by clinical sites that had the required CMR platform and software.^17,21^ A minimum of 2 data sets of short- and long-axis views were required to allow the core laboratory to select images of the highest quality. All gated data were displayed and reviewed by CMR core laboratory expert technologists to verify that LV boundaries were accurately demarcated. Short-axis images allowed determination of LVEF, using software developed by the CMR core laboratory at the University of Southern California (USC Cardio) and based on the Simpson method. Papillary muscles and trabeculations were considered to be part of the LV cavity. All short-axis data were reviewed by a technologist and adjusted manually, if necessary, to optimize accuracy of LV contour borders.

### Statistical Analysis

In this secondary study of the STICH trial, agreement of the core laboratory determinations of LVEF between modalities were assessed using 5 indices of variability: mean signed difference, mean absolute difference, Pearson correlation coefficient, Bland-Altman plots in which the mean of 2 measurements was plotted against the difference,^22^ and coverage probability.^23^ For determining LVEF by echocardiography, 3 methods (biplane, single plane, and visual estimation) were included in this comparison. For the coverage probability analysis, which is an assessment of the proportion of participants where a prespecified level of agreement is present, agreement was assumed to be present if the LVEF measures being compared were within 5% of each other. Because LVEF measures with SPECT performed after stress could be influenced by ischemia or stunning, Bland-Altman plots were repeated after exclusion of the 18% with SPECT LVEF assessed following stress. To assess the impact of nonsimultaneous imaging, the number of days between performances of various modalities was considered. Finally, the prognostic effect of the different measures of LVEF for association with all-cause mortality was assessed using Cox regression models. In each case, the relationship of LVEF with mortality was modeled using restricted cubic spline functions.^24^ The relative risk of every 5% LVEF increment, expressed as hazard ratio and 95% confidence interval, was also calculated using the Cox model. The longest available follow-up information was used for each patient.^16,25^ This was a 2-sided test with a *P* value less than .05 required for significance.

## Results

### Clinical Characteristics

The population included 2032 patients (95.1%) (mean [SD] age, 60.9 [9.6] years; 1759 [86.6%] male) of the 2136 patients in the STICH trial who had LVEF assessed by at least 1 imaging modality. A baseline echocardiographic assessment of LVEF was received by the core laboratory in 1978 patients. Of these, 30 (1.5%) were excluded (imaging was performed >90 days before or after randomization in 24 cases and images were unusable in 6 cases). The remaining 1948 patients constitute the population studied with echocardiography. Biplane Simpson method of determining LVEF was performed in 897 (46%) of the 1948 patients. Only 3 patients (<1%) received echo contrast. Gated SPECT images for assessment of LVEF were received by the core laboratory in 790 patients. Of these, 16 (2.0%) were excluded (imaging was performed >90 days before or after randomization in 14 cases and images were unusable in 2 cases). The remaining 774 patients constitute the population studied with SPECT. Cardiac magnetic resonance assessment of LVEF was performed and received by the core laboratory in 425 patients. Of these, 7 (1.6%) were excluded (imaging was performed >90 days before or after randomization in 6 cases and 1 patient with out-of-range LVEF measurement that could not be confirmed by the CMR core laboratory). The remaining 417 patients constitute the population studied with CMR. Although most patients had echocardiography, the population included 84 patients who had only gated SPECT or CMR. Thus, 1107 (57%) of the 1948 patients with echocardiographic determination of LVEF also had LVEF determined by a second modality. Characteristics of the patients who were evaluable are shown in Table 1. Patients with better-quality echocardiographic images were more likely to undergo imaging with SPECT or CMR. For those with excellent echocardiographic image quality, 42.3% had SPECT (42.5% were good, 35.6% were fair, and 29.7% were borderline; *P* < .001). For those with excellent echocardiographic image quality, 36.6% had CMR (21.1% were good, 18.2% were fair, and 15.6% were borderline; *P* < .001). Compared with those who had only imaging by echocardiography, patients who also had imaging by SPECT or CMR more often had prior myocardial infarction (79.9% vs 83.9%; *P* = .02), prior percutaneous coronary revascularization (9.9% vs 21.0%; *P* < .001), greater anterior akinesia or dyskinesia (45.3 [50.5%] vs 48.4 [26.7%]; *P* < .001), and lower New York Heart Association heart failure class (class I or II in 58% vs 61.6%; *P* < .001). Left ventricular ejection fraction was measured by all 3 modalities in 127 patients; these patients compared with those with LVEF measured by 1 or 2 modalities more often had prior myocardial infarction (93.7% vs 80.9%; *P* < .001), prior percutaneous coronary revascularization (34.6% vs 14.1%; *P* < .001), greater anterior akinesia or dyskinesia (57.5 [13.1%] vs 46.0 [40.3%]; *P* < .001), and lower New York Heart Association heart failure class (class I or II in 68.5% vs 56.7%; *P* = .02).

**Table 1.** Baseline Characteristics of 2032 Patients With Left Ventricular Ejection Fraction Data From Core Laboratories^a^^,^^b^

Characteristics	No. (%)		
	Patients With Echocardiographic EF (n = 1948)	Patients With SPECT EF (n = 774)	Patients With CMR EF (n = 417)
Age, mean (SD), y	60.9 (9.5)	61.2 (9.4)	61.0 (9.6)
Male	1687 (86.6)	667 (86.2)	359 (86.1)
BMI, mean (SD)	27.4 (4.6)	27.6 (4.4)	27.4 (4.5)
Hyperlipidemia	1271 (65.4)	515 (66.8)	300 (72.6)
Hypertension	1165 (59.8)	443 (57.2)	241 (57.8)
Current smoker	408 (21.0)	141 (18.2)	100 (24.0)
Diabetes	712 (36.6)	284 (36.7)	151 (36.2)
Peripheral vascular disease	292 (15.0)	122 (15.8)	51 (12.2)
Chronic renal insufficiency	152 (7.8)	60 (7.8)	26 (6.2)
Stroke	133 (6.8)	57 (7.4)	22 (5.3)
Myocardial infarction	1595 (81.9)	642 (82.9)	364 (87.3)
Previous CABG	56 (2.9)	18 (2.3)	10 (2.4)
Previous PCI	301 (15.5)	160 (20.7)	100 (24.0)
Atrial flutter or fibrillation	237 (12.2)	94 (12.1)	47 (11.3)
Current NYHA heart failure class			
I	200 (10.3)	80 (10.3)	35 (8.4)
II	915 (47.0)	429 (55.4)	201 (48.2)
III	757 (38.9)	240 (31.0)	156 (37.4)
IV	76 (3.9)	25 (3.2)	25 (6.0)
Anterior akinesia or dyskinesia, mean (SD), %	47.0 (39.2)	46.3 (29.0)	56.0 (14.6)
Study quality			
Excellent	71 (3.6)	250 (32.3)	334 (80.3)
Good	791 (40.6)	380 (49.1)	66 (15.9)
Fair	578 (29.7)	134 (17.3)	13 (3.1)
Borderline	508 (26.1)	10 (1.3)	3 (0.7)

Abbreviations: BMI, body mass index (calculated as weight in kilograms divided by height in meters squared); CABG, coronary artery bypass grafting; CMR, cardiovascular magnetic resonance; EF, ejection fraction; NYHA, New York Heart Association; PCI, percutaneous coronary intervention; SPECT, single-photon emission computed tomography.

^a^ Some patients have left ventricular ejection fraction from more than 1 core laboratory.

^b^ Continuous variables are presented as mean (SD) and categorical variables are presented as No. (%).

### Imaging

The median time interval between echocardiography and SPECT was 3.0 days (interquartile range, 1.0-9.0 days); between echocardiography and CMR was 2.0 days (interquartile range, 1.0-6.0 days); and between SPECT and CMR was 1.0 days (interquartile range, 1.0-5.0 days). All patients qualified for participation in the study based on LVEF of 35% or less as assessed by the recruiting site. Thus, in some patients, an alternative modality of determination of LVEF yielded a result of 35% or greater. The mean (SD) for the LVEF by the core laboratories for each modality and for the various echocardiographic methods and the numbers and percentages with LVEF of 35% or less for each modality are shown in Table 2.

**Table 2.** Data on LVEF According to Echocardiographic Method and Imaging Modality

Variables	Patients With Data by Modality, No. (%)	LVEF, Mean (SD), %	Patients With LVEF ≤35%, No. (%)
Echocardiographic EF	1948 (95.9)	29.0 (8.2)	1555 (79.8)
Echocardiographic biplane EF	897 (44.1)	28.7 (8.2)	709 (79.0)
Echocardiographic single-plane EF	725 (35.7)	29.2 (8.6)	552 (76.1)
Echocardiographic visual EF	1941 (95.5)	28.5 (7.8)	1679 (86.5)
SPECT EF	774 (38.1)	26.8 (8.3)	648 (83.7)
CMR EF	417 (20.5)	27.2 (10.8)	328 (78.7)

Abbreviations: CMR, cardiovascular magnetic resonance; EF, ejection fraction; LVEF, left ventricular ejection fraction; SPECT, single-photon emission computed tomography.

### Comparison of Imaging Modalities

Among 1948 patients who had LVEF assessed by echocardiography, 1437 (73.8%) of them had both visual estimate and quantitative LVEF. The mean absolute differences between LVEF as determined by quantitative vs visual echocardiographic methods were all less than 5% (mean absolute difference 2.7% for biplane and visual, 3.0% for single plane and visual, and 2.9% for biplane and single plane). The mean absolute differences of LVEF by echocardiography and by other modalities were all greater than 5% but were lowest when biplane echocardiography, rather than other echocardiographic methods, was used (data not shown). Mean absolute difference of LVEF between modalities was 5.9% for biplane echocardiography and SPECT (n = 385), 7.3% for CMR and biplane echocardiography (n = 204), and 5.9% for CMR and SPECT (n = 134). The variability measurements were similar in women and men (data not shown). When only data for studies performed within 3 days were considered, results were similar: mean absolute difference 5.8% for biplane echocardiography and SPECT (n = 213), 7.2% for CMR and biplane echocardiography (n = 126), and 5.9% for CMR and SPECT (n = 84). Even when only data for studies performed the same day were considered, results were similar: mean absolute difference 5.2% for biplane echocardiography and SPECT (n = 68 ), 8.6% for CMR and biplane echocardiography (n = 33), and 4.1% for CMR and SPECT (n = 10). When only data for studies rated as having excellent or good quality were included, mean absolute differences were again similar: 5.6% for biplane echocardiography and SPECT (n = 317), 7.3% for CMR and biplane echocardiography (n = 184), and 5.7% for CMR and SPECT (n = 116). For the 127 patients in whom LVEF was measured by all 3 modalities, mean absolute difference between modalities was 6.0% for echocardiography and SPECT, 6.8% for CMR and echocardiography, and 5.9% for CMR and SPECT.

Bland-Altman comparisons between modalities for determining LVEF are shown in the Figure. Limits of agreement between modalities were broad, ranging from 28.27% to 35.31%. Bland-Altman comparison with SPECT repeated after exclusion of LVEF assessed following stress with SPECT were similar (eFigure in the Supplement). The mean signed difference between LVEF measured by biplane and single-plane echocardiography was closest to 0, indicating no substantial overestimation or underestimation of LVEF by either echocardiographic method. The mean signed differences for LVEF between modalities were larger with wider 95% confidence intervals.

**Figure.**
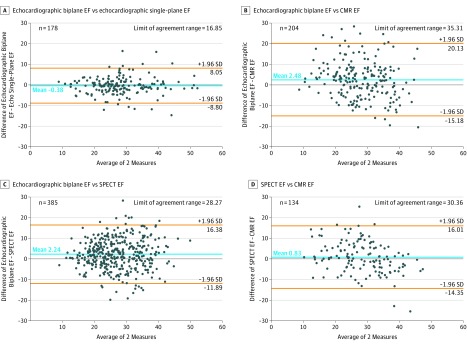
Bland-Altman Plots for Left Ventricular Ejection Fraction (EF) Plots are compared for biplane Simpson method and visual estimation for echocardiography (A), biplane Simpson method by echocardiography and gated single-photon emission computed tomography (SPECT) (B), biplane Simpson method by echocardiography and cardiovascular magnetic resonance (CMR) (C), and gated SPECT and CMR (D).

Correlations between LVEF as determined by quantitative vs visual echocardiographic methods (*r* = 0.898 for biplane vs visual, *r* = 0.876 for single plane vs visual, *r* = 0.874 for biplane and single plane) were higher than the correlations of LVEF assessed between different modalities (*r* = 0.601 for biplane echocardiography and SPECT, *r* = 0.660 for CMR and SPECT, and *r* = 0.493 for CMR and biplane echocardiography).

Left ventricular ejection fraction measurements were within 5% in 54.0% for biplane echocardiography and SPECT, 48.5% for SPECT and CMR, and 43.1% for biplane echocardiography and CMR. Using CMR as the standard and comparing it with SPECT and echocardiography as to whether there was intermodality agreement for LVEF greater than 35% is shown in Table 3. The results of the 4 variability indices comparing LVEF and end-systolic volume index for the 3 modalities are summarized in Table 4.

**Table 3.** Agreement and Disagreement for LVEF 35% or Greater According to Echocardiographic Method and Imaging Modality Using LVEF by CMR as the Standard

Comparison LVEF	No. of Patients With Both LVEFs	No. (%) of Patients					
		Both EF ≤35%	Both EF >35%	2 EFs Agreed	CMR EF ≤35% and Comparison EF >35%	CMR EF >35% and Comparison EF ≤35%	2 EFs Disagreed
Echocardiographic EF	377	243 (64.5)	37 (9.8)	280 (74.3)	54 (14.3)	43 (11.4)	97 (25.7)
Echocardiographic biplane EF	204	134 (65.7)	18 (8.8)	152 (74.5)	34 (16.7)	18 (8.8)	52 (25.5)
Echocardiographic single-plane EF	130	80 (61.5)	17 (13.1)	97 (74.6)	18 (13.9)	15 (11.5)	33 (25.4)
Echocardiographic visual EF	375	271 (72.3)	25 (6.7)	296 (78.9)	24 (6.4)	55 (14.7)	79 (21.1)
SPECT EF	134	90 (67.1)	19 (14.2)	109 (81.3)	10 (7.5)	15 (11.2)	25 (18.7)

Abbreviations: CMR, cardiovascular magnetic resonance; EF, ejection fraction; LVEF, left ventricular ejection fraction; SPECT, single-photon emission computed tomography.

**Table 4.** Variability Indices for LVEF and ESVI Measures Between Modalities

Pairwise Comparison of Core Laboratory LVEF or ESVI Measures	Mean Signed Difference	Mean Absolute Difference	Correlation Coefficient	Bland-Altman Limit of Agreement Range^a^	Coverage Probability^b^
Echocardiographic biplane EF vs SPECT EF, % (n = 385)	2.2	5.9	0.601	28.27	0.540
SPECT EF vs CMR EF, % (n = 134)	0.8	5.9	0.660	30.36	0.485
Echocardiographic biplane EF vs CMR EF, % (n = 204)	2.5	7.3	0.493	35.31	0.431
Echocardiographic biplane ESVI vs SPECT ESVI, mL/m^2^ (n = 386)	−12.8	20.8	0.821	97.92	0.332
SPECT ESVI vs CMR ESVI, mL/m^2^ (n = 134)	11.0	17.9	0.821	83.04	0.358
Echocardiographic biplane ESVI vs CMR ESVI, mL/m^2^ (n = 204)	−3.8	18.8	0.786	26.67	0.353

Abbreviations: CMR, cardiovascular magnetic resonance; EF, ejection fraction; ESVI, end-systolic volume index; LVEF, left ventricular ejection fraction; SPECT, single-photon emission computed tomography.

^a^ Bland-Altman limit of agreement range is between 1.96 standard deviation of the differences between the 2 imaging modalities. Approximately 95% of the differences are expected to fall within this range.

^b^ Coverage probability is the proportion of participants who fall within the prespecified acceptable paired absolute difference. For LVEF, the prespecified acceptable paired absolute difference was 5% or less. For ESVI, the prespecified acceptable paired absolute difference was 10 mL/m^2^.

### Prognostic Impact of LVEF According to Modality

Among the 2032 patients who had LVEF assessment, follow-up was available in all, although it was abbreviated in 32 cases (1.6%). The prognostic effect of LVEF by modality and outcome was assessed. During a mean (SD) follow-up of 5.0 (3.3) years, there were 966 deaths. Among 1948 patients who had echocardiographic LVEF assessment, the prognostic effect of LVEF by method (biplane, single plane, or visual estimation) was examined. Left ventricular ejection fractions measured by different methods were all statistically significant in terms of their prognostic value with respect to all-cause mortality. The HR (0.83-0.89) and the associated 95% confidence intervals for every 5% LVEF increment were all similar and below 1.00. This indicates increased LVEF was associated with decreased mortality risk, regardless which modality for determining LVEF was used (eTable in the Supplement).

## Discussion

Left ventricular ejection fraction refers to the fraction of LV end-diastolic volume ejected during systole. It is the most widely used measure of assessment for LV systolic function and is familiar to patients and clinicians. This is the first study to compare echocardiographic, CMR, and SPECT methods for determination of LVEF in a large, international, multicenter cohort of patients included in a clinical trial with extensive follow-up. In this population with LV dysfunction and CAD, we found that there was substantial variation among modalities for determination of LVEF even though these measures were made by specialized core laboratories, each of which followed specific plans for image analysis and measurements.^19,26^ Acquisition of data at 127 sites worldwide may have contributed to this variability. Moreover, variation was not predictable; there was no substantial overestimation or underestimation of LVEF by any modality relative to another.

Surprisingly, few studies have assessed the differences in LVEF as measured by different imaging modalities.^12,13,14,27^ Previous studies were predominantly single center, most with fewer than 100 participants, and most recent studies have focused on 3-dimensional or contrast echocardiography. These newer techniques were associated with improved reproducibility and agreement with CMR.^28,29^

Correlations between various methods of determination of LVEF by a single modality, echocardiography, were similar and better (*r* = 0.898 for biplane and visual estimation; *r* = 0.874 for single plane and biplane; and *r* = 0.876 for single plane and visual) than were correlations between different modalities, which ranged from *r* = 0.493 (for biplane echocardiography and CMR) to *r* = 0.660 (for CMR and SPECT). The good correlation between visual estimation and measurement of echocardiographic images is of interest; however, it should be noted that visual estimates were made by echocardiographic core laboratory staff, who had advanced training and extensive experience. Results might be worse with less experienced reviewers. Although the 3 echocardiographic methods assessed were well correlated, LVEF by biplane method of disks should be used when feasible as it correlated best with LVEF by other modalities.

Bland-Altman analysis showed no substantial overestimation or underestimation of LVEF by different modalities. Biplane quantitation with echocardiography averaged only 2.5% higher than CMR and 2.2% higher than gated SPECT, and SPECT was 0.8% higher than CMR. Limits of agreement were broad. Variability between modalities for measures of LV end-systolic volume index were also broad.

Variation of LVEF within 5% between modalities might be considered clinically acceptable. However, the percentage of observations that fell within a range of 5% ranged from 43% to 54% between different imaging modalities. Discordance between modalities as to whether LVEF was greater than 35% was present in about 20% to 25% of cases. Had core laboratory determination of LVEF been used for enrollment in the STICH trial, which required LVEF of 35% or less, many of these patients would have been ineligible.

The decision to perform SPECT or CMR was made by the clinical site. Patients with greater akinesia or dyskinesia were more likely to undergo CMR. Geometric abnormalities of the left ventricle may have contributed to the discordance between modalities for determining LVEF. In particular, echocardiographic biplane and single-plane methods rely on geometric assumptions, whereas SPECT and CMR methods assess the LV tomographically, without these assumptions. This may have contributed to the poor concordance of echocardiography and the other methods. Had patients with worse echocardiographic images been more likely to be referred for additional imaging, this might have accounted for a poorer correlation between echocardiography and other modalities. However, patients with better echocardiographic image quality more often underwent performance of additional imaging with SPECT or CMR.

The prognostic effect of LVEF by different echocardiographic methods and between modalities was also assessed. Although LVEF measurements from different methods and modalities have significant variations, analyses in this study indicate LVEF was strongly prognostic of all-cause mortality in the univariable Cox regression models no matter which method or modality was used. Because LVEF was determined by different modalities for different patients, direct comparison of the prognostic effect for LVEF by various modalities was not possible.

### Limitations

No gold standard exists for determining LVEF; agreement between modalities was assessed by various statistical methods. All patients had reduced LVEF. Correlations may have been better had the population included patients with a full spectrum of values. A broader spectrum of values of LVEF would be expected to be encountered in most clinical situations. Despite recommendations that LVEF be determined using biplane Simpson method, this was possible in only 897 of patients with echocardiography (46%). Ultrasonography contrast agents, recommended for use when imaging is suboptimal,^30^ have been shown to improve correlation with CMR but were used in only 3 patients.^14,31^ Three-dimensional echocardiographic imaging is increasingly being used for determination of LVEF and may improve the agreement between echocardiography and other methods, but it is not available on all ultrasonography systems and was not used in this study.^32^ Similarly, technology for SPECT and CMR also continues to be refined; such improvements may reduce differences between modalities. The SPECT LVEF measurements obtained from poststress studies may have been lower than rest studies in patients with extensive ischemia. However, even after exclusion of data in which SPECT LVEF was assessed following stress, results were similar with wide limits of agreement. Only a subset of patients was referred for SPECT and CMR; decisions about referral were made at local sites. Only 57% of patients who underwent echocardiography had LVEF determined by a second modality. Only 38% of patients included in the analysis had LVEF determined by SPECT, and only 20.5% of patients included in the analysis had LVEF determined by CMR. Since not all patients included in the STICH trial had LVEF determined by 2 different modalities, there may have been selection bias in choosing patients who had LVEF determined by a second imaging modality.

Assessments by different modalities were not simultaneous, and intervening medical therapy or ischemia may have affected LVEF. Left ventricular ejection fraction can be affected by changes in preload, afterload, and LV remodeling.^33^ Data regarding changes in pharmacologic therapy or blood pressure between tests were not available. The median time interval between studies was 3 days between echocardiography and SPECT studies, 2 days between echocardiography and CMR studies, and 1 day between SPECT and CMR studies. Correlations were not consistently improved when tests were performed within 3 days or on the same day. Reproducibility of LVEF determination may be affected by image quality,^34^ but correlations were not improved when only images of good or excellent quality were included.

## Conclusions

Although LVEF is a widely reported measurement and is the cornerstone of many treatment decisions, there is substantial variability in its measurement using different modalities, even when assessed by core laboratories. In this international study in which LVEF imaging was performed at 127 clinical sites using up to 3 widely used imaging modalities and LVEF was independently measured by core laboratories according to standard protocols, the variability in LVEF measurement exceeded 5% in about half of the patients. Variability was less for different methods of determining LVEF when a single imaging modality (echocardiography, in this case) was used. Longitudinal assessments of a given patient may best be accomplished using a single imaging modality.

The diagnostic and prognostic importance of LVEF as well documented in numerous studies is not disputed. Left ventricular ejection fraction by each modality was associated with mortality. However, variability in LVEF assessment by different imaging modalities should be considered in trial design and clinical management. Considering this variability, cut points in LVEF should not be the sole basis for decision making.

## Notes

Supplement. **eFigure.** Bland-Altman Plots for LVEF After Exclusion of SPECT LVEF Data When Obtained After Stress **eTable.** Prognostic Effect of LVEF Measures in Cox Regression Model
