# Supplementary material for: Variability in Ejection Fraction Measured By Echocardiography, Gated Single-Photon Emission Computed Tomography, and Cardiac Magnetic Resonance in Patients With Coronary Artery Disease and Left Ventricular Dysfunction
Source: JAMA Netw Open. 2018 Aug 31;1(4):e181456. doi: 10.1001/jamanetworkopen.2018.1456 (PMC6324278; doi:10.1001/jamanetworkopen.2018.1456)
Supplement: Supplement. — eFigure. Bland-Altman Plots for LVEF After Exclusion of SPECT LVEF Data When Obtained After Stress eTable. Prognostic Effect of LVEF Measures in Cox Regression Model [file jamanetwopen-1-e181456-s001.pdf]

Supplementary Online Content

Pellikka PA, She L, Holly TA, et al. Variability in ejection fraction measured by echocardiography, gated single-photon emission computed tomography, and cardiac magnetic resonance in patients with coronary artery disease and left ventricular dysfunction. *JAMA Netw Open*. 2018;1(4):e181456. doi:10.1001/jamanetworkopen.2018.1456

eFigure. Bland-Altman Plots for LVEF After Exclusion of SPECT LVEF Data When Obtained After Stress
eTable. Prognostic Effect of LVEF Measures in Cox Regression Model

This supplementary material has been provided by the authors to give readers additional information about their work.

© 2018 Pellikka PA et al. *JAMA Network Open*.

eFigure. Bland-Altman Plots for LVEF After Exclusion of SPECT LVEF Data When Obtained After Stress

A. Biplane Simpson's Method by Echocardiography and Gated SPECT

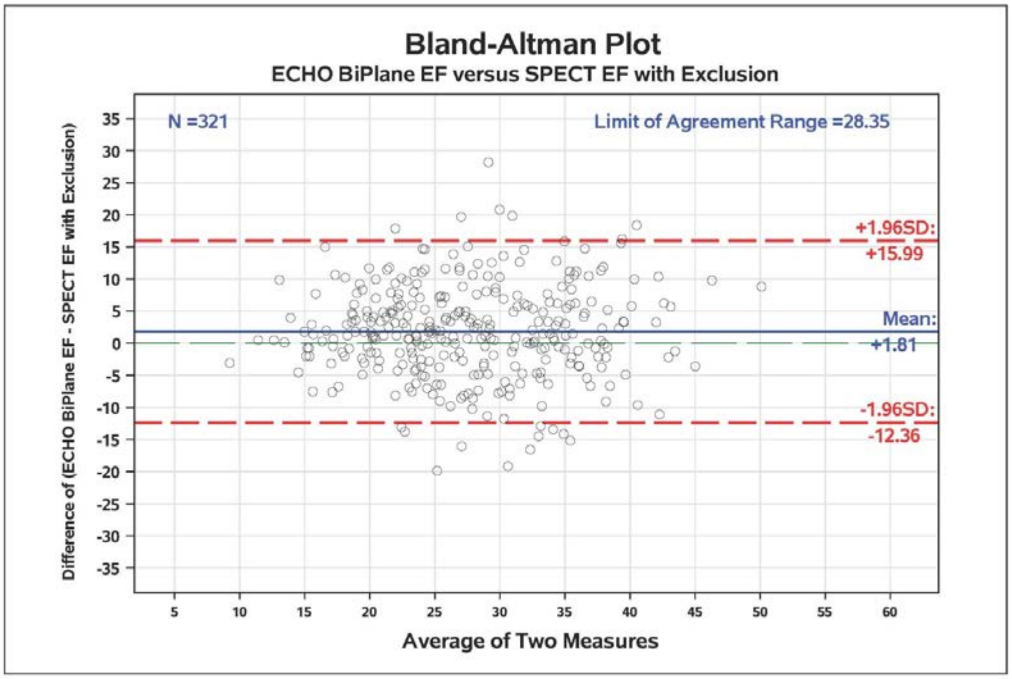

B. Gated SPECT and CMR

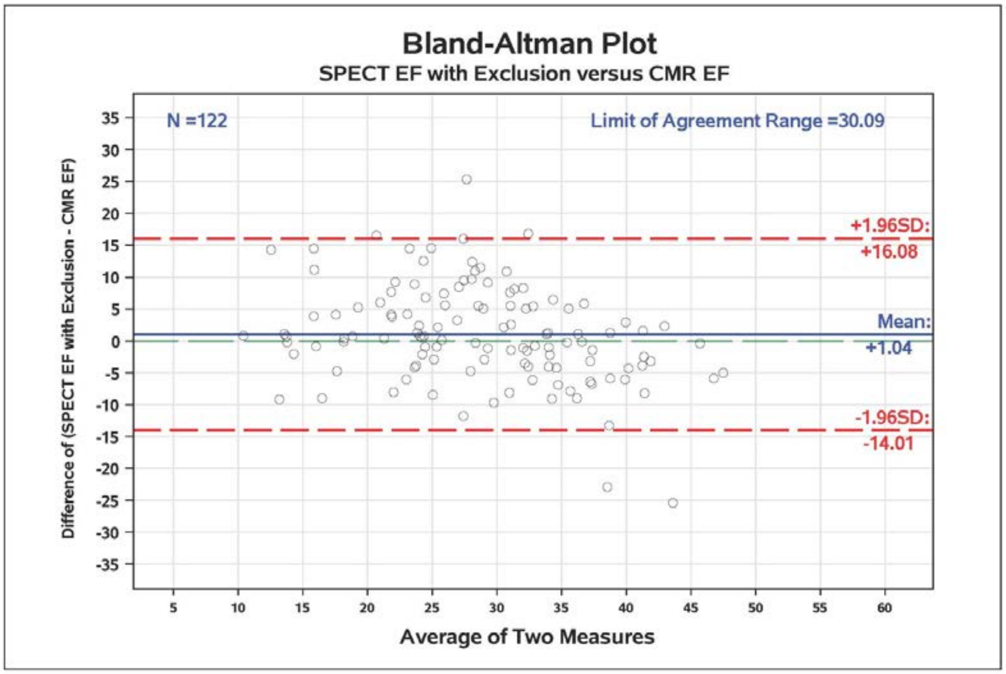

Plots are compared for A. Biplane Simpson's method by echocardiography and gated SPECT, B. Gated SPECT and CMR

© 2018 Pellikka PA et al. *JAMA Network Open*.

eTable. Prognostic Effect of LVEF Measures in Cox Regression Model

Variables	Number of Patients with LVEF Data by Modality	Number of Deaths	Likelihood Ratio Chi-square	Hazard Ratio (95% CI)[1]	*P* value
Echocardiographic EF	1948	924	59.42	0.86 (0.83, 0.90)	<0.001
Echocardiographic Biplane EF	897	434	44.32	0.83 (0.78, 0.88)	<0.001
Echocardiographic Single Plane EF	725	317	21.78	0.86 (0.81, 0.92)	<0.001
Echocardiographic Visual EF	1941	922	54.63	0.86 (0.82, 0.90)	<0.001
SPECT EF	774	391	37.04	0.83 (0.78, 0.88)	<0.001
CMR EF	417	159	10.92	0.89 (0.82, 0.96)	0.012

Note: Hazard ratio and (95% CI) for every 5% increment of LVEF.

© 2018 Pellikka PA et al. *JAMA Network Open*.
